# Associations between life’s essential 8 and non-alcoholic fatty liver disease among US adults

**DOI:** 10.1186/s12967-022-03839-0

**Published:** 2022-12-23

**Authors:** Lili Wang, Jiayi Yi, Xinli Guo, Xiangpeng Ren

**Affiliations:** 1 College of Medicine, Jiaxing University, No.899 Guangqiong Road, Jiaxing City, Jiaxing, 314001 Zhejiang Province China; 2 Department of Cardiology, Fuwai Hospital, Chinese Academy of Medical Sciences and Peking Union Medical College, National Center for Cardiovascular Diseases, Beijing, China

**Keywords:** NHANES, Cardiovascular health, Life’s Essential 8, NAFLD

## Abstract

**Background:**

Nonalcoholic fatty liver disease (NAFLD) is closely associated with Cardiovascular disease (CVD). We aim to examine the association of Life’s Essential 8 (LE8), the recently updated measurement of cardiovascular health (CVH), with the presence of NAFLD among US adults.

**Methods:**

This population-based cross-sectional study used data from the National Health and Nutrition Examination Survey in 2017–2018 and included adults 20 years or older. LE8 score (range 0–100) was measured according to American Heart Association definitions and was categorized into low (0–49), moderate (50–79), and high (80–100) CVH. NAFLD was determined by transient elastography measured hepatic steatosis in the absence of other liver diseases and excess alcohol use. Multivariable logistic and restricted cubic spline models were used to assess the associations.

**Results:**

Among 3588 participants included (weighted mean age, 48.0 years; 95% confidence interval [CI] 46.4–49.7 years), 1839 were female (weighted percentage, 51.6%; 95% CI 49.0–54.2%) and 1483 were determined to have NAFLD (weighted percentage, 36.5%; 95% CI 33.3–39.7%). The weighted mean LE8 score of the study population was 67.9 (95% CI 66.6–69.2). After the adjustment of potential confounders, higher LE8 scores were associated with reduced odds of NAFLD (odds ratio [OR] for per 10 score increase, 0.67; 95% CI 0.59–0.76) and a nonlinear dose–response relationship was observed. Similar patterns were also identified in the association of health behavior and health factor scores with NAFLD. The inversed association of LE8 score and NAFLD was significantly stronger among younger, Asian, and participants with higher education and income level.

**Conclusions:**

LE8 and its subscales scores were negatively associated with the presence of NAFLD in non-linear fashions. Promoting adherence to optimal CVH levels may be beneficial to reduce the burden of NAFLD as well as CVD.

**Supplementary Information:**

The online version contains supplementary material available at 10.1186/s12967-022-03839-0.

## Introduction

Non-alcoholic fatty liver disease (NAFLD) refers to a broad range of clinical and pathological findings which is characterized by excess fat accumulation in hepatocytes in the absence of excessive alcohol consumption or other competing causes for hepatic steatosis [1, 2]. It has become one of the most prevalent chronic liver diseases and affects 25% general population worldwide [3, 4]. NAFLD is recognized as the liver component of a collection of conditions that are associated with systematic metabolic dysfunction, including abdominal obesity, hypertension, atherogenic dyslipidemia, and insulin resistance, which are also well-established risk factors of cardiovascular disease (CVD) [5–7]. Increasing evidence indicates the presence of NAFLD is associated with an increased prevalence and incidence of CVD.

In 2010, the American Heart Association (AHA) proposed Life’s Simple 7 (LS7) as a measurement of cardiovascular health (CVH) to further improve the general population health [8]. Extensive subsequent evidence has proved the ideal CVH defined by LS7 was associated with greater CVD-free survival and total longevity and higher quality of life [9–12]. However, limitations of the original LS7 CVH score were also identified. Accordingly, the AHA recently updated the assessment tool for quantification of CVH namely “Life’s Essential 8” (LE8) [9]. LE8 is a more sensitive scoring system to inter-individual differences and highlights social determinants of health and psychological health for maintaining or improving CVH [13].

Given the close associations between NAFLD and the established CVD risk factors, promoting CVH may be an appropriate prevention and management strategy for reducing the burden of NAFLD. To date, several studies have indicated that optimal LS7 level was associated with decreased risk of incidents of NAFLD [14–17], while no study has investigated the association between the novel CVH construct and NAFLD. In this study, using the lasted available National Health and Nutrition Examination Surveys (NHANES) data, we aim to assess the association of LE8 and NAFLD in a nationally representative population of US adults.

## Methods

### Study design and participants

The NHANES is a periodic, cross-sectional health survey program using a stratified, multistage, and probability-cluster design to collect a nationally representative sample of non-institutionalized US civilians [18]. The National Center for Health Statistics (NCHS) administered the survey, and the institutional ethics review board of NCHS approved the study protocol. Written informed consent to participate was obtained from all participants. This cross-sectional analysis used data from the 2017–2018 NHANES cycles. This study followed the Strengthening the Reporting of Observational Studies in Epidemiology (STROBE) reporting guideline [19].

Of the 9254 participants from NHANES 2017–2018, 5569 participants aged 20 years or older were included. We excluded 304 participants without hepatic vibration-controlled transient elastography (VCTE) data. We further excluded participants having the following conditions: (1) elastography examination status was ineligible (n = 226), not performed (n = 136), or partial (n = 394); (2) serologic positivity for viral hepatitis (n = 70); (3) taking steatogenic medications (prednisone, tamoxifen, and methotrexate) for at least 3 months or more before the survey (n = 65); (4) excessive alcohol use defined as more than 2 or 3 standard alcoholic drinks per day on average for women or men, respectively (n = 121); (5) having missing data of CVH metrics (n = 665). The final study population included 3588 adult participants (Additional file 1: Figure S1).

### Demographic characteristics

Demographic characteristics were collected by questionnaires during the home interview. In this study, age was stratified into 3 strata: 20–39 years, 40–59 years, or ≥ 60 years. Race/ethnicity was categorized as non-Hispanic (NH) White, NH Black, NH, Asian, Hispanic, and Other. The poverty ratio was calculated as the ratio of monthly family income to poverty levels and categorized into 3 groups: < 1.3 (low income), 1.3–3.5 (middle income), and > 3.5 (high income). Education levels were categorized as high school graduate or less, some college, and college graduate or above. Marital status was categorized as coupled and single or separated.

### Measurement of LE8

LE8 scoring algorithm consists of 4 health behaviors (diet, physical activity, nicotine exposure, and sleep duration) and 4 health factors (body mass index [BMI], non-high-density-lipoprotein cholesterol, blood glucose, and blood pressure). Detailed algorithms for calculating the LE8 scores for each of the metrics to NHANES data have been previously published and can be found in Additional file 1: Table S1 [9, 13]. In brief, each of the 8 CVH metrics was scored ranging from 0 to 100 points. The overall LE8 score was calculated as the unweighted average of the 8 metrics. Participants with a LE8 score of 80–100 were considered high CVH; 50–79, moderate CVH; and 0–49 points, low CVH [9]. In this study, we used the same definition and cut-off points to measure and categorize health behavior and health factor scores to further investigate the association between LE8 subscales and NAFLD.

Diet metric was evaluated by the Healthy Eating Index (HEI) 2015 [20]. The components and scoring standards HEI–2015 were summarized in Additional file 1: Table S2. The dietary intakes of participants collected from two 24 h dietary recalls were combined with the United States Department of Agriculture (USDA) food patterns equivalents data to construct and calculate the HEI-2015 scores [21]. The simple HEI scoring algorithm method (by person) was used to compute the HEI-2015 score using an official SAS code provided by National Cancer Institute [22]. Self-report questionnaires collected physical activity, smoking and sleeping information, diabetes history, and medication history. Blood pressure, height, and weights were measured during the physical examination. The BMI was calculated as the weight in kilograms divided by the height in meters squared. Blood samples were collected and sent to central laboratories for the determination of blood lipids, plasma glucose, and hemoglobin A1c.

### Measurements and definition of NAFLD

Transient elastography examinations were performed for all participants aged 12 years and older in NHANES 2017–2018 cycle. A detailed protocol of NHANES transient elastography examinations has been described previously [23]. In brief, participants were examined to assess the controlled attenuation parameter (CAP) score and liver stiffness measurements using the FibroScan^®^ model 502 V2 Touch (Echosens, Waltham, MA). A complete examination was defined as 10 or more valid stiffness measurements, fasting time of at least 3 h, and liver stiffness interquartile range/median ≤ 30%. The median CAP was dichotomized using 285 dB/m as a threshold for liver steatosis diagnosis with optimum diagnostic performance (sensitivity of 80% and specificity of 77%) [24].

### Statistical analysis

Given the complex sampling design of NHANES, all analyses in this study accounted for sample weights, clustering, and stratification to generate nationally representative estimates. Categorical variables were presented as weighted percentages, and continuous variables as weighted means, with corresponding confidence intervals (CIs). Baseline characteristics were compared using the Rao-Scott chi-square test for categorical variables and unadjusted linear regressions for continuous variables. Age-standardized prevalence estimates and 95% CIs were calculated for each category of CVH level.

Survey-weighted multivariable logistic regressions were used to investigate the independent association of CVH with the risks of NAFLD after the adjustment of potential demographic confounders and obesity (defined as BMI ≥ 30 kg/m^2^). Restricted cubic spline regression was applied to examine the potential nonlinear relationships between the LE8 score and its subscales score with NAFLD. Nonlinearity was tested using the likelihood ratio test.

To examine subpopulations susceptible to demographic-related disparities, stratified analyses were performed by sex, age strata, race/ethnicity, poverty ratio, and education levels. The P values for the production terms between LE8 scores and the stratified factors were used to estimate the significance of interactions. We also performed sensitivity analysis by (1) using propensity score matching to correct the confounding factors (age, sex, race/ ethnicity, obesity, aspartate aminotransferase, alanine aminotransferase, γ-glutamyl transferase, and triglycerides) between the NAFLD and non-NAFLD groups; (2) excluding participants having self-reported histories of cardiovascular diseases (including coronary heart disease, angina, heart attack, and stroke; n = 379) to assess the robustness of our findings.

Statistical tests were 2-sided, and statistical significance was set at P < 0.05. All analyses were performed with SAS version 9.4 (SAS institute, Cary, NC) using the “SURVEY” procedures and R software, version 4.2.0 (R Core Team, Vienna, Austria).

## Results

### Baseline characteristics

A total of 3588 participants aged 20 years or older were included. Baseline characteristics of the study population were summarized by the category of NAFLD status in Table 1. The weighted mean age of the study participants was 48.0 years (95% CI 46.4–49.7 years), and 1839 were female (weighted percentage [WP] 51.6%, 95% CI 49.0–54.2%). The mean LE8 score was 67.9 (95% CI 66.6–69.2) and the weighted percentages of low, moderate, and high CVH were 9.1% (95% CI 7.5–10.6%), 69.2% (95% CI 66.0–72.3%), and 21.7% (95% CI 18.3–25.2%) separately. 1483 participants were diagnosed with NAFLD (WP 36.5%, 95% CI 33.3–39.7%). Compared to those without NAFLD, participants with NAFLD were older and more likely to be male, had lower education levels, and were coupled. The LE8 scores were higher in participants without NAFLD, while the nicotine exposure and sleep duration scores had no significant difference among the two groups (Table 1).

**Table 1:** Baseline Characteristics of the study population^*^

	No.^†^	Overall (n = 3588)	Non NAFLD (n = 2105)	NAFLD (n = 1483)	*p* value
Age, years		48.0 (46.4–49.7)	46.1 (44.3–47.9)	51.4 (49.7–53.0)	< 0.01
Age strata					< 0.01
20–39	1112	36.6 (32.6–40.6)	42.7 (38.0–47.4)	26.1 (21.9–30.5)	
40–59	1140	34.6 (31.2–38.0)	31.4 (27.7–35.1)	40.3 (34.4–46.2)	
≥ 60	1336	28.8 (24.5–33.0)	25.9 (21.6–30.3)	33.7 (27.6–39.7)	
Female	1839	51.6 (49.0–54.2)	55.8 (53.1–58.5)	44.4 (39.6–49.2)	< 0.01
Race/ethnicity					< 0.01
NH White	1284	64.3 (59.0–69.6)	63.7 (57.9–69.5)	65.3 (59.4–71.2)	
NH Black	798	10.3 (6.9–13.7)	12.0 (8.3–15.6)	7.3 (4.4–10.3)	
Hispanic	839	15.5 (11.5–19.6)	14.0 (10.3–17.7)	18.2 (13.1–23.3)	
NH Asian	481	5.2 (3.5–6.9)	5.5 (3.5–7.5)	4.7 (2.9–6.5)	
Other	186	4.7 (3.4–6.1)	4.8 (3.2–6.5)	4.5 (2.4–6.5)	
Poverty ratio					0.26
< 1.3	852	18.8 (17.1–20.5)	19.4 (17.6–21.3)	17.6 (14.9–20.3)	
1.3–3.5	1301	35.5 (31.3–39.7)	34.1 (29.7–38.6)	37.9 (32.1–43.7)	
> 3.5	1028	45.7 (41.1–50.3)	46.4 (41.7–51.2)	44.5 (38.2–50.9)	
Education levels					< 0.01
High school or less	1461	36.5 (32.5–40.5)	34.3 (29.3–39.3)	40.3 (35.7–45.0)	
Some college or associates degree	1203	30.9 (27.7–34.2)	29.4 (25.3–33.5)	33.6 (30.1–37.1)	
College graduate or above	919	32.5 (26.7–38.4)	36.3 (30.0–42.6)	26.1 (19.9–32.2)	
Marital status					< 0.01
Coupled	2163	63.5 (60.2–66.9)	59.5 (55.0–64.1)	70.5 (66.4–74.7)	
Single or separated	1423	36.5 (33.1–39.8)	40.5 (35.9–45.0)	29.5 (25.3–33.6)	
LE8 scores (out of 100 possible points)					
LE8 score	/	67.9 (66.6–69.2)	72.0 (70.4–73.5)	60.8 (59.5–62.1)	< 0.01
HEI-2015 diet score	/	38.3 (35.1–41.5)	39.9 (36.1–43.7)	35.5 (32.2–38.8)	0.02
Physical activity score	/	77.5 (75.6–79.4)	79.5 (76.7–82.3)	74.0 (70.3–77.7)	0.04
Nicotine exposure score	/	75.3 (72.8–77.8)	75.0 (71.9–78.0)	75.8 (73.4–78.3)	0.51
Sleep health score	/	87.0 (85.7–88.3)	87.2 (85.5–88.9)	86.6 (84.8–88.4)	0.64
Body mass index score	/	56.6 (53.2–60.0)	69.1 (66.0–72.1)	34.8 (30.7–39.0)	< 0.01
Blood lipids score	/	67.0 (64.3–69.7)	71.5 (68.8–74.3)	59.0 (55.8–62.3)	< 0.01
Blood glucose score	/	74.7 (73.5–76.0)	80.6 (79.2–82.1)	64.5 (62.0–67.0)	< 0.01
Blood pressure score	/	66.9 (64.9–68.8)	73.0 (70.8–75.2)	56.1 (52.6–59.6)	< 0.01
Cardiovascular health^‡^					< 0.01
Low	424	9.1 (7.5–10.6)	5.1 (3.6–6.5)	16.1 (13.5–18.7)	
Moderate	2506	69.2 (66.0–72.3)	64.3 (60.1–68.4)	77.7 (75.3–80.1)	
High	658	21.7 (18.3–25.2)	30.7 (26.2–35.2)	6.2 (3.7–8.8)	

*NAFLD* non-alcoholic fatty liver disease, *LE8* life’s essential 8, *HEI* healthy eating index, *CVH* cardiovascular health

^*^Data were presented as weighted percentages or means (95% confidence intervals)

^†^Numbers of each stratum may not add up to the total population due to missing data

^‡^Low CVH was defined as a LE8 score of 0 to 49, moderate CVH of 50–79, and high CVH of 80–100

### LE8 score and NAFLD

**Fig. 1:**
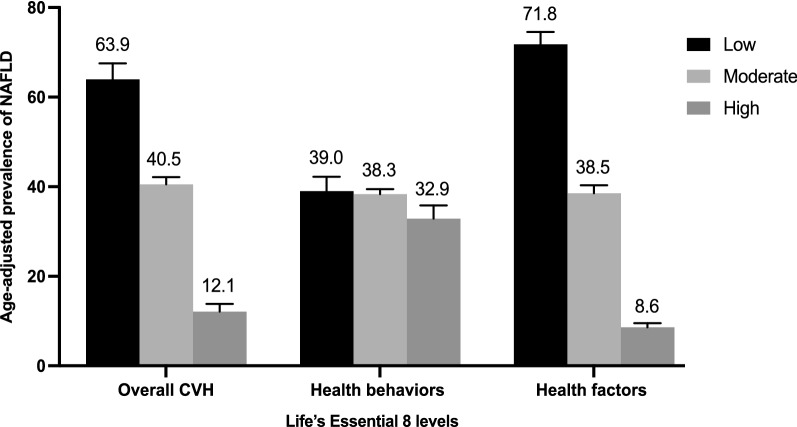
Age-adjusted prevalence of non-alcoholic fatty liver disease (NAFLD) in different levels of Life’s Essential 8 scores. Numbers at the top of the bars represent the weighted percentage. Bar whiskers represent the 95% confidence level

**Fig. 2:**
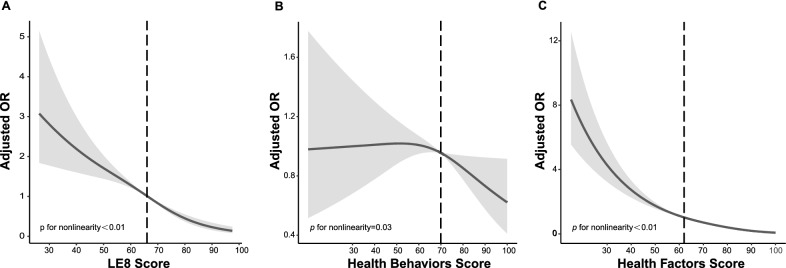
Dose–response relationships between Life’s Essential 8 scores (**A**), Health Behavior score (**B**), Health Factors Score(**C**), and non-alcoholic fatty liver disease (NAFLD). ORs (solid lines) and 95% confidence levels (shaded areas) were adjusted for age (as a continuous variable), sex, race/ethnicity, obesity; poverty ratio (as a continuous variable), education level, and marital status. Vertical dotted lines indicate the minimal threshold for the beneficial association with estimated OR = 1. *OR* odds ratio, *LE8* Life’s Essential 8

The age-adjusted prevalence of NAFLD was significantly lower in participants with high CVH (12.1%, 95% CI 8.7–15.5%) than in those with moderate (40.5%, 95% CI 37.5–43.6%) and low CVH (63.9%, 95% CI 56.9–71.0%; Fig. 1). After multivariable adjustment, compared with the low CVH group, the odds ratios (ORs) of NAFLD were 0.53 (95% CI 0.41–0.70) in the moderate CVH group and 0.19 (95%CI 0.12–0.30) in the high CVH group, respectively. OR for every 10 scores increase in LE8 score was 0.67 (95%CI 0.59–0.76) in association with NAFLD (Table 2). A nonlinear association was observed between the LE8 score and NAFLD (*p* < 0.01 for nonlinearity; Fig. 2A). The minimal threshold for the beneficial association was 66 scores (estimate OR = 1).

**Table 2:** Association of the Life’s Essential 8 scores with non-alcoholic fatty liver disease (NAFLD)

	Univariable model		Multivariable model 1^*^		Multivariable model 2^†^	
	OR (95% CI)	*p* value	OR (95% CI)	*p* value	OR 95%CI	*p* value
LE8 score						
Low (0–49)	1 (Reference)	/	1 (Reference)	/	1 (Reference)	/
Moderate (50–79)	0.38 (0.28–0.51)	< 0.01	0.55 (0.40–0.75)	< 0.01	0.53 (0.41–0.70)	< 0.01
High (80–100)	0.06 (0.04–0.11)	< 0.01	0.20 (0.11–0.34)	< 0.01	0.19 (0.12–0.30)	< 0.01
Per 10 points increase	0.53 (0.47–0.60)	< 0.01	0.68 (0.60–0.77)	< 0.01	0.67 (0.59–0.76)	< 0.01
Health behaviors score						
Low (0–49)	1 (Reference)	/	1 (Reference)	/	1 (Reference)	/
Moderate (50–79)	0.98 (0.75–1.28)	0.58	0.90 (0.68–1.18)	0.43	0.95 (0.73–1.22)	0.93
High (80–100)	0.77 (0.55–1.09)	0.33	0.85 (0.59–1.22)	0.77	0.91 (0.61–1.34)	0.64
Per 10 points increase	0.93 (0.88–0.99)	0.02	0.95 (0.89–1.00)	0.05	0.97 (0.90–1.04)	0.23
Health factors score						
Low (0–49)	1 (Reference)	/	1 (Reference)	/	1 (Reference)	/
Moderate (50–79)	0.25 (0.19–0.34)	< 0.01	0.36 (0.26–0.49)	< 0.01	0.34 (0.25–0.47)	< 0.01
High (80–100)	0.04 (0.02–0.05)	< 0.01	0.09 (0.06–0.14)	< 0.01	0.09 (0.05–0.14)	< 0.01
Per 10 points increase	0.53 (0.49–0.58)	< 0.01	0.61 (0.55–0.67)	< 0.01	0.60 (0.54–0.66)	< 0.01

*OR* odds ratio, *CI* confidence interval, *LE8* life’s essential 8

^*^Adjusted for age (as a continuous variable), sex, race/ethnicity, and obesity status

^†^Additionally adjusted for poverty ratio (as a continuous variable), education levels, and marital status

### Health behavior score and NAFLD

The age-adjusted prevalence of NAFLD was not significantly different among the three levels of health behavior groups (Fig. 1; *p* = 0.15). In the multivariable regression analysis, moderate and high health behavior groups were not significantly associated with NAFLD. OR for every 10 scores increase in health behavior score was 0.97 (95% CI, 0.90–1.04) in association with NAFLD (Table 2). A nonlinear association was observed between the health behavior score and NAFLD (*p* = 0.03 for nonlinearity) (Fig. 2B). The minimal threshold for the beneficial association was 70 scores (estimate OR = 1).

### Health factors and NAFLD

The age-adjusted prevalence of NAFLD was significantly lower in participants with high health factors (8.6%, 95% CI 6.8–10.4%) than in those with moderate (38.5%, 95% CI 35.0–42.0%) and low health factors (71.8%, 95% CI 66.4–77.5) (Fig. 1). After multivariable adjustment, compared with the low health factors group, the ORs of NAFLD were 0.34 (95% CI 0.25–0.47) in the moderate health factors group and 0.09 (95%CI 0.05–0.14) in the high health factors group, respectively. OR for every 10 scores increase in health factors score was 0.60 (95%CI 0.54–0.66) in the association with NAFLD (Table 2). A nonlinear association was observed between the health factors score and NAFLD (*p* < 0.01 for nonlinearity) (Fig. 2C). The minimal threshold for the beneficial association was 62 scores (estimated OR = 1).

### Subgroup and sensitivity analysis

**Fig. 3:**
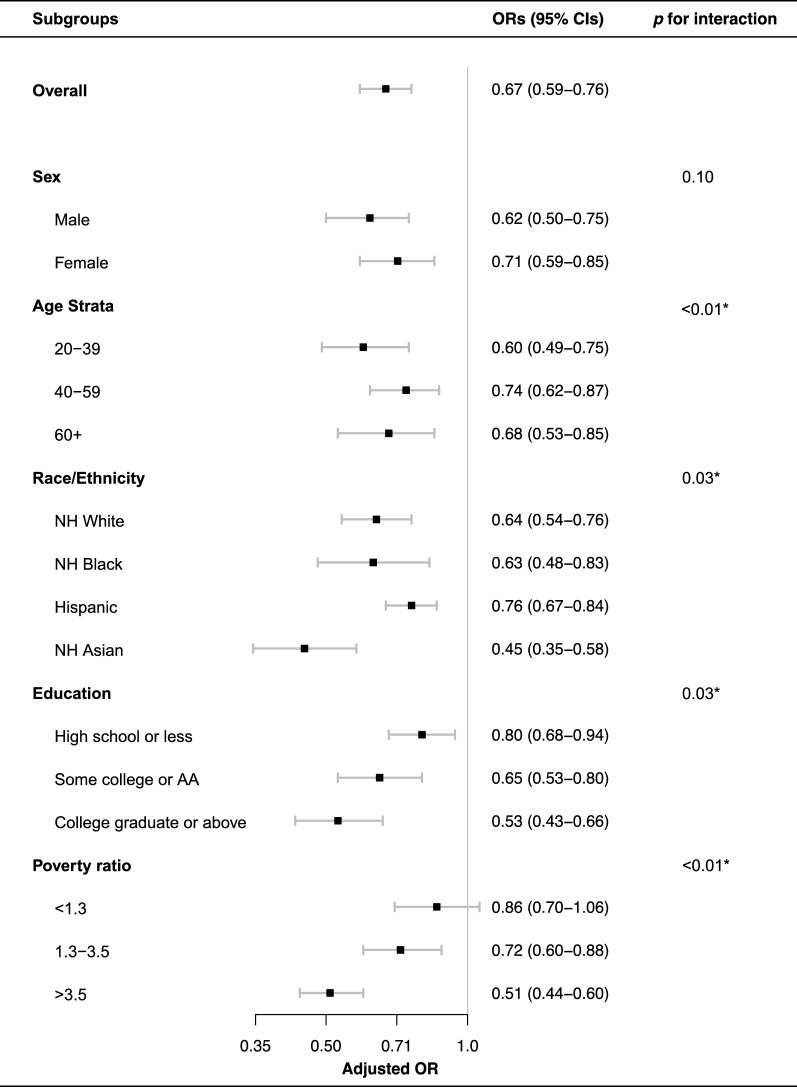
Subgroup analysis of the association of the Life’s Essential 8 scores and the presence of non-alcoholic fatty liver disease (NAFLD). ORs were calculated as per 10 scores increase in LE8 score. Each stratification was adjusted for age (as a continuous variable), sex, race/ethnicity, obesity; poverty ratio (as a continuous variable), education level, and marital status. *OR* odds ratio, *CI* confidence interval, *NH* non-Hispanic, *AA* associate degree

The results of subgroup analyses are presented in Fig. 3. The LE8 score was negatively associated with NAFLD in all subgroups. We found significant interactions between LE8 score and age, race, education levels, and poverty ratio with the presence of NAFLD (*p* < 0.05 for interaction). The inverse association between LE8 score and NAFLD appeared stronger in younger participants (aged 20–39 years; OR for per 10 scores increase, 0.60; 95% CI 0.49–0.75), the NH Asian individuals (OR for per 10 scores increase, 0.4045; 95% CI 0.35–0.58), the participants with college graduate or above education background (OR for per 10 scores increase, 0.53; 95% CI 0.43–0.66), and the high-income population (poverty ratio > 3.5; OR for per 10 scores increase, 0.51; 95% CI 0.44–0.60). The results were generally robust in sensitivity analyses (Table 3). The distribution of characteristics and propensity scores of the matching study population (n = 1822) were summarized in Additional file 1: Table S3, Figures S2 and S3. The association of LE8 score and health factors score with NAFLD remained significant after propensity score matching (OR for per 10 score increase in LE8 score, 0.88; 95% CI 0.82–0.95; OR for per 10 score increase in health factors score, 0.85; 95% CI 0.81–0.90) and excluding participants with CVD history (OR for per 10 score increase in LE8 score, 0.62; 95% CI 0.53–0.71; OR for per 10 score increase in health factors score, 0.57; 95% CI 0.52–0.62;). The health behaviors score remains insignificantly associated with NALFD in two sensitivity analyses (*p* > 0.05).

**Table 3:** Sensitivity analysis of the association of the Life’s Essential 8 scores with non-alcoholic fatty liver disease (NAFLD)

	Propensity score matching^*^		Excluding CVD history participants	
	OR (95% CI) ^*^	*p* value	OR (95% CI) ^†^	*p* value
LE8 score				
Low (0–49)	1 (Reference)	/	1 (Reference)	/
Moderate (50–79)	0.73 (0.55–0.95)	0.02	0.39 (0.27–0.55)	< 0.01
High (80–100)	0.49 (0.33–0.72)	< 0.01	0.12 (0.06–0.26)	< 0.01
Per 10 points increase	0.88 (0.82–0.95)	< 0.01	0.62 (0.53–0.71)	< 0.01
Health behaviors				
Low (0–49)	1 (Reference)	/	1 (Reference)	/
Moderate (50–79)	0.94 (0.73–1.21)	0.63	0.88 (0.61–1.29)	0.92
High (80–100)	1.03 (0.78–1.35)	0.86	0.80 (0.49–1.32)	0. 40
Per 10 points increase	1.01 (0.96–1.06)	0.84	0.93 (0.84–1.03)	0.10
Health factors				
Low (0–49)	1 (Reference)	/	1 (Reference)	/
Moderate (50–79)	0.72 (0.59–0.88)	< 0.01	0.31 (0.22–0.43)	< 0.01
High (80–100)	0.42 (0.30–0.58)	< 0.01	0.03 (0.01–0.09)	< 0.01
Per 10 points increase	0.85 (0.81–0.90)	< 0.01	0.57 (0.52–0.62)	< 0.01

*OR* odds ratio, *CI* confidence interval, *LE8* Life’s Essential 8

^*^Matching for age, sex, race/ ethnicity, obesity status, aspartate aminotransferase, alanine aminotransferase, γ-glutamyl transferase, and triglycerides

^†^Adjusted for age (as a continuous variable), sex, race/ethnicity, obesity; poverty ratio (as a continuous variable), education level, and marital status

## Discussion

In this nationally representative cross-sectional study, we found inverse dose–response associations between the LE8 score and its health behavior and health factors subscales with NAFLD in US adults. Subgroup analysis indicated that the negative association between LE8 score and NAFLD was stronger among younger, Asian, and high-income participants. The associations remained significant after excluding participants with CVD history.

To our knowledge, several studies have assessed the association between LS7 and NAFLD. A U.S.-based multiethnic cohort revealed that a more favorable LS7 level was associated with a lower prevalence of NAFLD [14]. In a Korean cohort, higher LS7 scores were associated with a decreased risk of incident NAFLD as well as the regression of existing NAFLD among younger adults [17]. In a cross-sectional study from Northern China, the prevalence rates of NAFLD were inversely associated with LS7 summary score quartiles [25]. Our finding is consistent with the current knowledge that NAFLD is inversely associated with CVH levels. However, as the predecessor of LE8, LS7 features definitions may not be able to reflect the full scope of health behaviors and practices in the current situation. In addition, research has revealed limitations in how the metrics are quantified [9]. The CHV definitions of LS7 were categorized into ideal, intermediate, and poor CVH for each component. This definition is less sensitive to interindividual differences and is unable to be used to assess dose–response effects.

The present study added notable evidence of the relationship between CVH and NAFLD by using LE8 as the definition of CVH. We found that the ORs in the association of health factors score with NAFLD decreased sharply in the lower range of the value. The benefits plateaued and then persisted unchanged across the higher values. While the trend was reversed in the association between health behavior score and NAFLD. ORs remain unchanged in the lower range of health behaviors score value and decrease quickly in the higher range. The saturation effect was observed in the association of health factors with NALFD while not in health behavior which indicates a more rigorous standard of health behavior might be preferable. In addition, the association between LE8 score and NAFLD was found to be stronger among younger, Asian, and higher education and income participants. These findings reveal that LE8 enhanced methods for quantification of CVH to increase the sensitivity of scoring to inter-individual differences in both individuals and populations. In addition, these results also highlight the disparity in the potential beneficial value of CHV components and population-level approaches should be implemented to promote CVH.

Although the mechanism between LE8 and NAFLD remains unclear, studies have demonstrated that NAFLD is significantly associated with metabolic syndrome and healthy lifestyles which are intrinsic health factors and health behaviors metrics of LE8 [26–30]. Obesity, a well-established risk factor of cardiovascular disease, is correlated with the expansion of adipose tissue, which leads to dysfunction and death of adipocytes. In the setting of adipose dysfunction, macrophages infiltrate into the adipose tissue and induce inflammation that promotes insulin resistance [2]. In the context of insulin resistance, inappropriate lipolysis and the compromised fat-storing ability of adipose tissue result in the release of free fatty acids into the circulation, which then becomes available for uptake by the liver and overwhelms its metabolic capacity [1, 2]. Both adipose tissue dysfunction and hepatic de-novo lipogenesis were considered as major NAFLD-inducing factors. Inflammation also plays an important role both in CVD and NAFLD. It was reported that Bisphenol A, an endocrine disruptor, could increase the relative risk of both CVD and NAFLD by inducing pro-inflammatory activities and overproduction of interleukin 1β (IL-1β) and IL-6 [31]. Systematic inflammation and circulating cyto- and chemokines including C-reactive protein, IL-6, IL1β, and TNFα fuel CVD through endothelial dysfunction, altered vascular tone, enhanced plaque formation, and coagulation [32]. Increased circulating inflammation markers are also associated with NAFLD. Blood levels of IL-6 were increased in accordance with the histological severity of non-alcoholic steatohepatitis. It is therefore not surprising a composite score of all the LE8 metrics is associated with NAFLD.

Several potential pharmacologic therapies for NAFLD have been studied. Vincenzo et.al found that Empagliflozin, a sodium-glucose cotransporter 2 inhibitor, reduced hepatic and cardiac inflammation in doxorubicin-treated mice which could be effective to reduce the presence and progression of both NAFLD and cardiovascular diseases [33]. However, most evidence remains experimental and there is no licensed drug for NAFLD until now. Therefore, the lifestyle modification approach remains the foundation of NAFLD management [34–37]. However, most previous studies have focused on individual factors in relation to NAFLD and all-around lifestyle recommendations for NAFLD patients are lacking. LE8 is a comprehensive and easily applicable assessment tool in clinical settings to promote adherence to healthy behaviors and ideal health factors. Our study extends the range of health outcomes associated with a beneficial role of ideal CVH metrics in NAFLD in addition to CVD and indicates that adherence to ideal CVH metrics may be an appropriate prevention and management strategy for reducing the burden of NAFLD as well as other chronic diseases including CVD.

The main strength of this study is the use of a large nationally representative sample of US adults which allows the findings to be generalized to a broader population. In addition, we addressed the dose–response relationship between CVH and NAFLD and identified the minimal threshold for the beneficial association. Several potential limitations should also be considered. Firstly, health behavior metrics assessments were based on self-report questionnaires which are subject to measurement errors. Secondly, we used the VTCE result as the diagnosis standard of hepatic steatosis. However, for its well-known limitations, it is neither practical nor feasible to perform liver biopsies on a vast population. Considering VTCE is a sensitive measurement of liver fat [38], it could be regarded as a satisfactory assessment tool in a large population-based epidemiologic study setting. Finally, although we controlled for several potential confounders, the nature of the cross-sectional study design precludes us from concluding causality and temporality between CVH and NAFLD risk.

## Conclusions

In this nationally representative sample of US adults, higher LE8 and its subscales scores were independently associated with the lower presence of NAFLD in non-linear fashions. Furthermore, the strength of the association between LE8 score and NAFLD differed within the study population. Our study results indicate a potential beneficial role of LE8 as a feasible and effective approach for promoting hepatic health. Further research on the longitudinal and causality association of LE8 and NAFLD risk is needed.

## Supplementary Information

**Additional file 1: Figure S1. **Flow chart of the screening process for the selection of the study population. **Table S1.** Definition and scoring approach for the American Heart Association’s Life’s Essential 8 score. **Table S2.** Healthy Eating Index-2015 Components & Scoring Standards^1^. **Table S3.** Characteristics of the matched study population. **Figure S2.** Distribution of variables standardized mean difference before and after matching. **Figure S3.** Distribution of propensity score before and after matching.

## Data Availability

The National Health and Nutrition Examination Survey dataset is publicly available at the National Center for Health Statistics of the Center for Disease Control and Prevention (https://www.cdc.gov/nchs/nhanes/index.htm).

## Abbreviations

NAFLD: Non-alcoholic fatty liver disease
CVD: Cardiovascular disease
AHA: American Heart Association
LS7: Life’s simple 7
CVH: Cardiovascular health
LE8: Life’s essential 8
NHANES: National health and nutrition examination surveys
NCHS: National Center for Health Statistics
STROBE: Strengthening the reporting of observational studies in epidemiology
VCTE: Vibration-controlled transient elastography
NH: Non-hispanic
BMI: Body mass index
HEI: Healthy eating index
USDA: United States Department of Agriculture
CAP: Controlled attenuation parameter
CI: Confidence interval
WP: Weighted percentage
OR: Odds ratio
IL: Interleukin
